# Dosimetric comparison of fixed field dynamic IMRT and VMAT techniques in simultaneous integrated boost radiotherapy of prostate cancer

**DOI:** 10.1097/MD.0000000000032063

**Published:** 2022-12-16

**Authors:** Haitao Sun, Ning Wang, Guosen Huang, Xiangping Liu

**Affiliations:** a Department of Radiotherapy Room of Medical Imaging Department, Zhongshan Hospital of Traditional Chinese Medicine, Zhongshan, China.

**Keywords:** intensity modulated radiotherapy, prostate cancer, simultaneously integrated boost, volumetric modulated arc therapy

## Abstract

High-risk prostate cancer can take advantage of the combination of hypofractionated radiotherapy and pelvic conventional fraction radiotherapy. The comparison between fixed field dynamic IMRT and VMAT techniques can provide suggestions for clinical treatment. We selected 10 high-risk prostate cancer patients who received radiotherapy at the cancer center of Sun Yat-sen University from January 2016 to December 2019. The targets contained in prostate, seminal vesicles and pelvic lymph nodes. With the same prescription and optimized parameters, 9F, single-arc (1ARC) and double-arc (2ARC) treatment plans were developed. The dose distribution of the targets, OAR, MU, treatment time and gamma pass ratios of dose verification was compared. The D_2%_ (69.37 ± 0.89) Gy, D_50%_ (66.92 ± 0.63) Gy, HI (0.09 ± 0.02), and CI (0.83 ± 0.05) of PTV1 in 9F were slightly better than those of 1ARC which were (71.13 ± 1.21) Gy, (68.50 ± 0.76) Gy, (0.12 ± 0.02), (0.74 ± 0.07), except D_98%_, the difference was significant (*P* < .05). All dosimetry indices of PTV1 in 9F and 2ARC were close and have no significant differences (*P* > .05). The V_95%_ (99.45 ± 0.78)% of PTV2 in 9F was slightly better than that in 1ARC (99.35 ± 1.28)%. The difference was significant (*P* < .05). All dosimetry indices of PTV2 in 9F and 2ARC were close and the difference was not significant (*P* > .05). The D_mean_ of the bladder and the V67.5 Gy of rectum between all three plans were similar. The D_mean_ of left and right femoral in 1ARC and 2ARC were lower than that in 9F, and the difference was significant (*P* < .05). Other dosimetry indices of OARs in 9F were lower than those in 1ARC and 2ARC, and much lower than 1ARC. The difference was significant (*P* < .05). Mean monitor units in 1ARC and 2ARC were fewer by 70.0% and 67.2% in comparison with 9F. The treatment mean time in 1ARC and 2ARC was shorter by 81.7% and 61% in comparison with 9F. Verification pass ratios of γ (3%/3 mm) were 97.8% (9F), 98.9% (1ARC) and 99.4% (2ARC) respectively. The difference was significant (*P* < .05). Compared with IMRT, VMAT improved delivery efficiency noticeably. Two arcs provided comparable tumor dosimetry coverage, but performed worse in dose sparing for bladder, rectum and small bowel. The IMRT plan was preferable to VMAT in prostate cancer simultaneous integrated boost radiotherapy.

## 1. Introduction

Prostate cancer is the most common noncutaneous cancer in men, with 1,212,653 cases in 2019 in China alone. Treatment of localized prostate cancer has been proven by clinical trial including hypo-fractionation radiation therapy (RT) dose escalation with Elective Nodal Irradiation and androgen deprivation therapy (ADT) combined with RT.^[1–5]^ With the development of radiotherapy intensity modulated radiation therapy (IMRT), it has been widely used in prostate cancer. Radiation therapy related toxicity is associated with high total RT dose, shorter recovery time, and the volume of neighboring organs at risk (OAR) normal tissues (rectum, bowel, and bladder) ever in prostate-only RT.^[6,7]^ In recent years, with the development of radiotherapy technology, IMRT has replaced 3DCRT as the most common method of radiation therapy for prostate cancer for its conformable dose distribution which can reduce normal tissue toxicity.^[1,3]^ The most common method for IMRT delivery for prostate cancer involves^[5–9]^ fixed gantry positions with computer-generated, sliding-window multi-leaf collimator positions to modulate the dose to the prostate.^[8–10]^ A more recent IMRT technique named volume modulated arc therapy (VMAT) involves gantry rotation around the prostate with 1 to 4 arcs while the X-Ray beam is on. In VMAT technique the dose-rate varies while the gantry is moving around. Palma et al reported that the most favorable equivalent uniform doses and lowest doses to organs at risk were achieved with variable dose-rate VMAT, which was statistically significantly better than 5-field, IMRT for rectal and femoral head endpoints and better than constant dose-rate VMAT for most bladder and rectal endpoints.^[11–13]^ Multiple groups have observed that VMAT reduces beam-on time and the radiation dose relative to 7 to 9 field IMRT.

According to NCCN guidelines, doses of 75.6 to 79.2 Gy in conventional fractions to the prostate are appropriate for patients with low-risk cancers. For patients with intermediate or high-risk disease, doses up to 81.0 Gy provide improved PSA-assessed disease control. For radical radiotherapy, patients with low-risk cancer should not receive pelvic lymph node irradiation or ADT, patients with intermediate-risk cancer may be considered for pelvic lymph node irradiation and 4 to 6-month neoadjuvant/concomitant/adjuvant ADT. Patients with high-risk cancers are candidates for pelvic lymph node irradiation and the addition of neoadjuvant/concomitant/adjuvant ADT for a total of 2 to 3 years.^[14]^ The target area of this study includes prostate, seminal vesicle and pelvic lymph nodes. The studies were irradiated simultaneously; the former group is treated with conventional segmented irradiation of pelvic cavity and prostate, and then with local prostate boost, the latter group is also treated with conventional segmented irradiation.^[2,14]^ In this way, patients are treated longer, which is more expensive. We need to make two plans for one patient, which increases the workload of doctors and physicists. At present, several published studies have been shown that the effect of increasing single dose or reducing the total dose is similar to that of conventional fractionation irradiation and the toxicity is acceptable. Meanwhile, the second edition of NCCN guidelines for prostate cancer in 2014 also included the content of hypo-fraction radiotherapy for prostate cancer (2.4–4 Gy/fraction, treatment period of 4–6 weeks). Hypo-fraction can not only shorten the treatment time and reduce the medical cost, but also improve the efficiency of therapeutic resources. The combination of conventional segmented irradiation of the pelvic cavity and the hypo-fraction radiotherapy of the prostate will be more beneficial for the patients. In this paper, we compared the difference of dosimetry and treatment efficiency of the IMRT and VMAT technology which designed through Varian treatment plan system.

## 2. Materials and Methods

### 2.1. Patient selection and CT simulation

We selected 10 patients with high-risk prostate cancer who received radiotherapy at Sun Yat-sen University Cancer Center at 2017-01-04, aged 62 to 78 years, median age 70 years. All of those patients didn’t go through a radical prostatectomy. Planning CT scans were performed at 5 mm slice thickness using a dedicated helical CT scanner, from the upper abdomen to 5 cm below the Ischia tuberosities after immobilization with knee and feet support immobilization devices. CT images were transferred to the inverse treatment planning system through network. Patients were instructed to have a comfortably full bladder and an empty rectum of CT acquisition and before each treatment. The bladder was full to make the small intestine move downwards to reduce the volume of small intestine irradiation.

### 2.2. Contouring and planning

The clinical target volume (CTV). CTV1 was identified as the entire prostate and seminal vesicle (if involved); CTV2 included the whole prostate and seminal vesicle (if not involved). Plan target volume (PTV1) was generated by adding anisotropic 0.5 cm margin to the CTV1 apart from posteriority where 0.3 cm margin was added (to decrease prostate-rectal interface dose). PTV2 was 0.7 cm anisotropic expansion of CTV2 except posteriorly (0.5–0.7 cm) being dependent on rectal fullness. The prescribed dose of PTV1 was 67.5 Gy in 25 daily fraction and PTV2 was 47.5 Gy in 25 daily fraction. Contouring of the OAR followed the RTOG pelvic normal tissue contouring guidelines.^[15]^ The rectum was exposed from the level of the ischial tuberosities to the rectosigmoid flexure. The healthy bladder was contoured; femoral heads were delineated to the level of the ischial tuberosities. The bowel was contoured as the entire volume of peritoneal space to within 1 cm of the cranial margin of the nodal PTV. Treatment planning system is Varian Eclipse (version 10.1); the radiotherapy equipment is Varian Trilogy linear accelerator with 120 Multi-leaf Collimator. The isocenter accuracy is 5 mm. Three intensities modulated treatment plans were designed for each case using 6MV X-Ray, that was fixed field dynamic intensity modulation nine-field (9F), VMAT single-arc (1ARC) and double-arc (2ARC), respectively. The maximum dose rate was at the rate of 600 MU/min. Among them, for IMRT, 9F beams were treated using a dynamic multi-leaf collimator and the radiation directions was 160°, 120°, 80°, 40°, 0°, 320°, 280°, 240°, 200° respectively; For VMAT, the 1ARC adopts a single arc of 179° to 181° anticlockwise rotation and for the 2ARC, the first arc rotated from 179° anticlockwise to 181°, than the second arc rotated clockwise from 181° to 179°. During the rotation of gantry, the step length of the subfield segment is 2°. There was all adopted the simultaneous integrated boost technology. The minimum allowable dose of the PTV was 93% of the prescribed dose and the maximum allowable dose in the PTV was 115% of the prescribed dose. At least 95% of the PTV received > 95% of the prescribed dose. For OAR, bladder V_55Gy_ < 30%, V_67.5Gy_ < 10%; rectum V_55Gy_ < 30%, V_67.5Gy_ < 10%; left and right femoral head V_40Gy_ < 5%. The small intestine D_max_ < 50 Gy. The dose constraint and optimization parameters of the three plans were the same ones, and the dose calculation grid size was 2.5 mm, using the AAA dose algorithm.^[12]^

### 2.3. Plan quality evaluation

For the sake of convenience, the three plans were normalized after the completion. At least 95% of the PTV1 received > 95% of the prescribed dose. According to the ICRU83 report,^[16]^ the dose distribution in the target area was evaluated with the maximum dose D_2%_, the minimum dose D_98%_ and the median dose D50%, conformability index (CI) and homogeneity index (HI) are introduced to evaluate the planned dose distribution, where HI = (D_2%_ − D_98%_)/ D_50%_, CI = (TVRI × TVRI)/(TV × VRI)(D_X%_ is the dose received by X% of the target volume, TVRI represents the target volume within the prescription isodose volume, TV is the volume of the target area PTV, and VRI is the volume enclosed by 95% of the prescription dose line). The smaller the HI value is, the better the uniformity of the dose distribution in the target area is. The value of CI is between 0 and 1, which represent the ideal situation that the target volume coincides exactly with the treatment volume. If CI equals zero that represents a plan in which there is not any overlap between the two volumes. Dose constraints were also evaluated for each plan, V50Gy, V55Gy, V60Gy, V67.5Gy and D_mean_ were used to evaluate rectum and bladder, V40Gy and D_mean_ were used to evaluate left and right femoral head, and D_max_ and D_mean_ were used to evaluate small intestine. D_mean_ was the average dose received by OAR, and D_max_ was the maximum point dose hosted by OAR. Note the number of subfields, MU and effective treatment time (the time from the beginning of the beam out to the end of beam out after the completion of patient positioning), and compare the results of each execution parameter of the 3 groups. The semiconductor three-dimensional detector array dose verification system ArcCHECK was utilized to conduct dose verification for all plans. The maximum dose point was taken as the standard, and the dose threshold was set to 10%. The DTA and gamma pass rates of each plan were compared under the standard of 3%/3 mm.

### 2.4. Statistical method

SPSS19.0 software (Zhongshan, China) was used for statistical analysis. The Shapiro–Wilk test was used to test the difference of paired data of each index of treatment plan (9F and 1ARC, 9F and 2ARC). If it is in line with the normal distribution, *t* test was used to test the results of 9F and 1ARC, 9F and 2ARC, and Wilcoxon rank sum test was used to test the results of non-normal distribution (*P* < .05). The difference was statistically significant, and the data results were expressed as mean ± standard deviation ($\bar{x}$ ± s).

## 3. Results

### 3.1. Dosimetry comparison of target area

**Figure 1.**
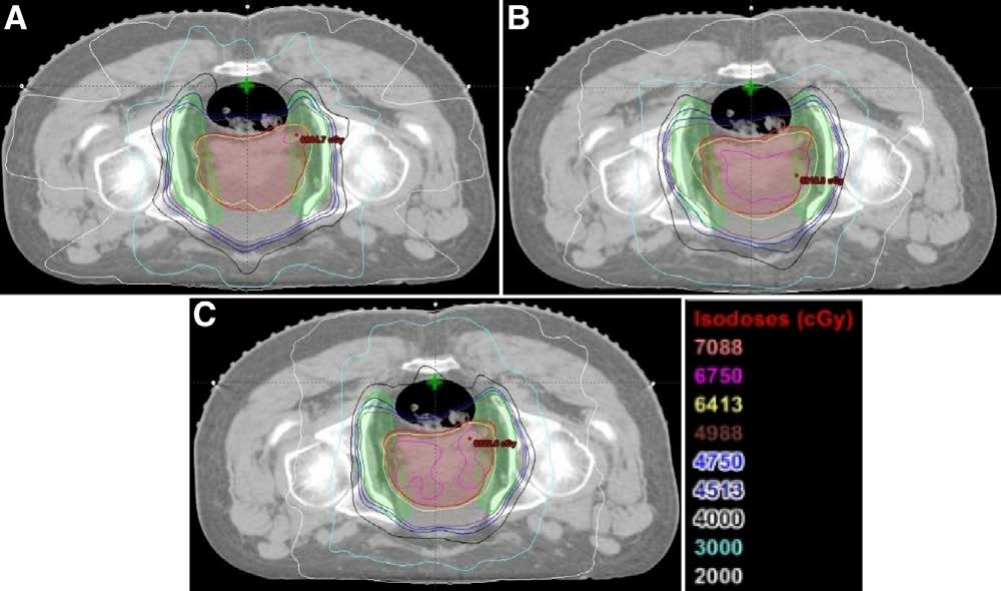
(A) Plan 9F, (B) Plan 1ARC, and (C) Plan 2ARC.The red line is PTV1, the green line is PTV2, and the other color lines are isodose lines. 1ARC = single-arc, 2ARC = double-arc, PTV = plan target volume.

The mean volume of PTV (including PTV1 and PTV2) was 1153.2 ± 146.3 cm^3^ (869.7–1409.0 cm^3^), which was large and complex. Figure 1 shows the isodose distribution on axial images of the three plans. PTV1 showed as a red line, PTV2 showed as a green line. PTV1 was inside PTV2. The isodose lines were displayed on an absolute dose scale, isodose levels increased by 20 Gy to 70.88 Gy gradients were shown. As dosimetry comparison of 9F with 1ARC and 2ARC is shown in Table 1: For PTV1, 9F plans were similar to 2ARC, there was no statistical significance (*P* > .05). And the D_98%_ of the three plans indices did not show any obvious difference. The D_2%_ and D_50%_ of 9F and 2ARC were closer to the prescription dose, and the HI and CI were slightly better than 1ARC, and the differences were statistically significant (*P* < .05). For PTV2, V_95%_ of 9F was better than 1ARC, the difference was statistically significant (*P* < .05), but D_98%_ has no statistical significance (*P* > .05), 9F and 2ARC has no statistical significance (*P* > .05).

**Table 1:** Dosimetric comparison of 9F with 1ARC and 2ARC target areas ($\bar{x}$ ± s).

Parameter	9F	1ARC	*t*/*z*	*P*	2ARC	*t*/*z*	*P*
PTV1							
D_2%_/Gy	69.37 ± 0.89	71.13 ± 1.21	−4.865	.001	69.15 ± 0.79	−0.561	.575*
D_98%_/Gy	63.18 ± 0.55	63.03 ± 0.31	0.86	.412	63.31 ± 0.20	−0.965	.360
D_50%_/Gy	66.92 ± 0.63	68.50 ± 0.76	−6.598	.000	67.20 ± 0.55	−1.730	.118
HI	0.09 ± 0.02	0.12 ± 0.02	−3.406	.008	0.09 ± 0.02	1.406	.193
CI	0.83 ± 0.05	0.74 ± 0.07	3.866	.004	0.79 ± 0.08	1.850	.097
PTV2							
V_95%_/%	99.45 ± 0.78	99.35 ± 1.28	2.549	.031	99.33 ± 0.75	0.448	.888*
D_98%_/Gy	46.13 ± 0.65	45.70 ± 1.24	0.959	.363	46.02 ± 6.14	0.442	.669

*z* is non-parametric test statistics.

1ARC = single-arc, 2ARC = double-arc, 9F = nine-field, CI = conformability index, HI = homogeneity index, PTV = plan target volume.

* Non-normal distribution data.

### 3.2. Dosimetry comparison of OAR

The mean results of OARs for dosimetry comparison of 9F with 1ARC and 2ARC plans are listed in Table 2. The three kinds of plans all met the clinical need. For rectum V_67.5Gy_, 9F was lower than 2ARC, but the difference was not statistically significant (*P* > .05); for bladder D_mean_, there was no statistically significant difference between the three groups (*P* > .05); for left and right femoral head D_mean_, 1ARC and 2ARC were lower than 9F, the difference was statistically significant (*P* < .05). Other OAR parameters evaluation, the 9F plans were lower than 1ARC and 2ARC, and the difference was statistically significant compared with 1ARC (*P* < .05).

**Table 2:** Dosimetric comparison of 9F with 1ARC and 2ARC plans for organs at risk ($\bar{x}$ ± s).

Parameter	9F	1ARC	*t*/*z*	*P*	2ARC	*t*/*z*	*P*
Rectum							
V_50Gy_/%	19.78 ± 3.55	28.2 ± 1.45	−8.949	.000	25.83 ± 0.81	−2.983	<.001
V_55Gy_/%	15.02 ± 3.14	21.19 ± 1.88	−10.851	.000	19.90 ± 1.41	−5.885	<.001
V_60Gy_/%	10.48 ± 2.39	15.08 ± 2.36	−7.606	.000	14.07 ± 1.83	−6.771	<.001
V_67.5Gy_/%	0.99 ± 1.21	3.91 ± 2.40	−4.324	.002	1.72 ± 1.90	−1.262	.207*
D_mean_/%	44.63 ± 1.00	47.21 ± 2.21	−4.339	.002	45.96 ± 1.45	−3.708	.005
Bladder							
V_50Gy_/%	28.97 ± 5.21	45.11 ± 12.55	−2.803	.005*	37.54 ± 4.96	−4.011	.003
V_55Gy_/%	18.56 ± 2.64	24.73 ± 1.85	−6.923	.000	20.81 ± 3.06	−3.011	.015
V_60Gy_/%	13.66 ± 2.97	16.64 ± 3.34	−5.415	.000	14.46 ± 2.76	−3.863	.004
V_67.5Gy_/%	0.88 ± 0.85	6.25 ± 2.53	−5.619	.000	3.09 ± 1.28	−3.946	.003
D_mean_/Gy	48.81 ± 2.00	47.99 ± 3.72	0.908	.387	47.55 ± 3.00	1.932	.085
Left femoral head							
V_40Gy_/%	1.8 ± 0.74	3.39 ± 1.01	−4.235	.002	2.49 ± 0.74	−2.697	.024
D_mean_/Gy	26.43 ± 3.54	23.68 ± 3.94	2.814	.020	23.22 ± 2.81	3.980	.003
Right femoral head							
V_40Gy_/%	1.75 ± 0.74	3.37 ± 0.86	−4.313	.002	2.44 ± 0.89	−2.531	.032
D_mean_/Gy	26.34 ± 3.27	22.05 ± 3.54	5.449	.000	22.73 ± 3.58	4.739	.001
Small intestine							
D_max_/Gy	44.05 ± 7.63	45.99 ± 9.07	−2.983	.018	45.18 ± 7.79	−2.379	.045

*z* is non-parametric test statistics.

1ARC = single-arc, 2ARC = double-arc, 9F = nine-fields.

* Non-normal distribution data.

### 3.3. Comparison of radiotherapy efficiency and dose

Compared with 9F, 1ARC, and 2ARC the monitor units (MUs) were reduced 70.0% and 67.2%, accounting for 9% and 18% of the number of subfields, respectively. The average delivery time of the 9F, 1ARC, and 2ARC plans was 449.3 ± 29 seconds, 82.2 ± 0.8 seconds, and 175.0 ± 0.9 seconds, respectively, decreased by 81.7% and 61%, relative to the 9F. The gamma and DTA pass rate for these plans exhibited was to be measured using the ArcCHECK detector along with ion chamber measurements. The average pass rate was higher than 95% under the standard of 3%/3 mm, and 1ARC and 2ARC were slightly higher than 9F. The differences of the above evaluation indicators were statistically significant (*P* < .05), as showed in Table 3.

**Table 3:** Comparison of 9F with 1ARC and 2ARC treatment efficiency and dose validation ($\bar{x}$ ± s).

Parameter	9F	1ARC	*t/z*	*P*	2ARC	*t*/*z*	*P*
Monitor units	1794.8 ± 155.2	534.5 ± 76.5	37.009	<.001	588.3 ± 155.0	22.308	<.001
Segment number	1907.5 ± 94.8	178.0 ± 0.0	57.685	<.001	356.0 ± 0.0	51.748	<.001
Treatment time/s	449.3 ± 29.0	82.2 ± 0.8	31.290	<.001	175.0 ± 0.9	22.747	<.001
DTA (3%/3 mm)	96.2 ± 1.2	97.9 ± 0.5	−2.201	.028*	99.1 ± 0.4	−2.201	.028*
γ (3%/3 mm)	97.8 ± 0.6	98.9 ± 0.4	−5.329	.003	99.5 ± 0.3	−9.463	<.001

*z* is non-parametric test statistics.

1ARC = single-arc, 2ARC = double-arc, 9F = nine-fields.

* Non-normal distribution data.

## 4. Discussion

Volumetric modulated arc therapy is widely used in clinical; there are many studies at home and abroad comparing its difference with the intensity modulated radiation therapy, including dosimetry, treatment efficiency and dose validation pass rate. A number of previous studies have shown that in head and neck tumors and esophageal cancer, the dose distribution of VMAT is equal to or slightly better than that of IMRT, which can greatly shorten the treatment time and improve the treatment efficiency. But, the previously reported target structure is often relatively simple. Research of Guckenberger and Bortfeld shows that VMAT may not provide enough intensity modulation for more complex targets.^[13,14,17–20]^ In order to obtain a shorter treatment time, single arc VMAT may over sacrifice the quality of dose distribution; increasing the number of subfields or rotating arcs can improve the quality of dose distribution; however, the treatment time will increase correspondingly.

In the comparative study of radiotherapy techniques for prostate cancer, different conclusions were drawn according to the target areas of different structures and shapes. In studies only including prostate or prostate and seminal vesicle, Bedford et al^[13]^ have shown that 1ARC VMAT had better PTV coverage and less OAR exposure dose than 5-field IMRT; Boylan et al have shown that 1ARC VMAT can better protect OAR compared with 5-field IMRT, but the PTV coverage was worse; other reports had shown that VMAT and IMRT have similar dose distribution, and with the number of fixed fields increases, the dose distribution of IMRT will gradually be equal to or even better than VMAT. Bijina et al^[21]^ used sequential irradiation to compare the dose distribution and treatment efficiency of IMRT with a single arc and double arc VMAT. Primary planning target volume contained prostate, seminal vesicles, and pelvic lymph node with a margin. The results showed that IMRT could protect bladder, rectum and small intestine, and had similar HI and CI to 2ARC, slightly better than 1ARC. For the second course plan, 2ARC and IMRT had similar dose distribution. The results of our study are similar to Yoo et al: the target area coverage of IMRT is no less than or slightly better than 1ARC, similar to 2ARC; except for D_mean_ of left and right femoral head and D_mean_ of bladder, other parameters of OAR are better than 1ARC and 2ARC, and significantly better than 1ARC, which is better protection of the bladder, rectum and small intestine. In addition, those plans were normalized such that the prescription dose covered at least 95% of the PTV, after which one plan with D5 of PTV ≤ 110% and better OAR sparing was selected for each technique. As shown in Figure 1, IMRT will be exposed to relatively lower fluoridization of 20 Gy and 30 Gy, and the smoothness of the dose curve will be worse. Compared with IMRT, double arc VMAT can significantly reduce the radiation dose of OAR, which is different from this result; the reason may be that the IMRT utilized in this study is 5F and the number of subfields is less. In general, the more field shots, the better the result.

The biggest advantage of VMAT is to greatly shorten the treatment time. This study shows that compared with IMRT, the average treatment time of 1ARC and 2ARC shorten by 81.7% and 61%, and also reduce the number of MUs by 70.0% and 67.2% respectively, which can reduce the loss of the treatment machine. Those results are similar to the results reported by Bijina et al,^[21]^ but Quan et al^[9]^ used AIP algorithm on Pinnacle v9.0 system to compare with VMAT and IMRT, VMAT had 30% more MUs than 8-field IMRT, but the treatment time was reduced 3 minutes. In addition, the three-dimensional dose validation results of three groups plans in this study meet the clinical requirements (γ ≥ 90%). Although VMAT plan is more complex than IMRT plan and involves more parameters (collimator angle, multi-leaf grating, dose rate, gantry rotation speed) in the process of treatment implementation, the measurement results of VMAT plan are better than IMRT. This may be because ArcCHECK is cylindrical for the phantom, the subfield in VMAT is smaller, and the angle difference of probe dose response is smaller, which makes it more suitable for the measurement of VMAT plan.^[18,22,23]^

In conclusion, under the condition that the prescription dose covered at least 95% of the PTV standard, whether using IMRT or VMAT technology in simultaneous integrated boost radiotherapy for prostate cancer can satisfy the need of OAR and have a good dose verification pass rate. Compared with the IMRT plan, VMAT plan can reduce the treatment time significantly and improve the treatment effectively; 2ARC plan has similar target coverage, but the protection of bladder, rectum and small bowel is worse; the less the number of VMAT arcs, the worse the OAR protection. With the number of arcs increases the quality of plan improves, but at the same time, the number of MUs and treatment time also increases. The results of this study show that for the complex target structure including prostate, seminal vesicle and pelvic lymph node drainage area, the use of IMRT technology can significantly improve the quality of planning and can better protect the OAR and is more suitable for the simultaneous integrated boost radiotherapy of prostate cancer pelvic radiation prevention. However, considering that the sample size used in this study is small the results need to be further verified by expanding the sample size. The plan designer needs to compare the advantages and disadvantages of VMAT and IMRT first with a larger sample size for cases with different target size or structure, weigh the gains and losses, and finally select a more appropriate treatment technology.

## 5. Conclusion

Compared with IMRT, VMAT noticeably improved delivery efficiency, with two arcs provided comparable tumor dosimetric coverage, performed worse in dose sparing for bladder, rectum and small bowel. The IMRT plan was better than VMAT in prostate cancer radiotherapy using a simultaneous integrated boost.

## Author contributions

All authors designed the study. NW and GH collected clinical data, HS and XL performed testing and interpreted the results. All authors helped in drafting and editing the manuscript. All authors read and approved the submission of the final version of the manuscript.

**Conceptualization:** Ning Wang, Guosen Huang.

**Data curation:** Haitao Sun, Ning Wang.

**Formal analysis:** Haitao Sun, Ning Wang.

**Writing – original draft:** Haitao Sun, Xiangping Liu.

**Writing – review & editing:** Guosen Huang.
